# Juvenile male rats form preferences based on strain when playing in groups but not in pairs

**DOI:** 10.3389/fnbeh.2025.1617178

**Published:** 2025-07-10

**Authors:** Jackson R. Ham, Diya Jaiswal, Renata Waner-Mariquito, Sergio M. Pellis, E. J. Marijke Achterberg

**Affiliations:** ^1^Department of Neuroscience, Canadian Centre for Behavioural Neuroscience, University of Lethbridge, Lethbridge, AB, Canada; ^2^Section Animals in Science and Society, Neurobiology of Behaviour Group, Department of Population Health Sciences, Faculty of Veterinary Medicine, Utrecht University, Utrecht, Netherlands

**Keywords:** play fighting, rough-and-tumble play, strain differences, social behavior, social conditioned place preference, ultrasonic vocalizations

## Abstract

Like many young mammals, juvenile rats engage in rough-and-tumble play. Play occurs naturally both in wild and laboratory rats, making it a suitable, ethologically relevant behavior to investigate. In the laboratory, rats are typically housed and tested in dyads, despite living in large colonies in the wild. Consequently, when tested in the lab, rats do not have a choice of partners and are instead paired with whomever the researcher selects. Given that both the amount and style of play rats engage in varies considerably depending on the strain of rat being studied, we tested whether rats select play partners based on strain. To do so, juvenile male Long Evans (LE) focal rats (*n* = 8) were subjected to three play contexts: (1) group play; (2) dyadic play; and (3) social conditioned place preference. During group play, the LE subject rats were given the choice to play with an LE, a Sprague Dawley (SD), or Fischer 344 rat (F344), simultaneously. During dyadic play, focal rats played one-on-one with an LE, SD, or F344 partner. Finally, the rats were conditioned to a context and a social stimulus, with the context either being paired with an LE (preferred stimulus) or F344 (unpreferred stimulus) partner. We found that, when given a choice in a group setting, LE focal rats prefer to play with same-strain partners over both SD and F344 partners. However, when playing under dyadic conditions (i.e., with an assigned partner), LE rats played with each strain equally. Finally, in the socially conditioned place preference test, we found that the focal rats formed preferences for a particular enclosure, but not for the strain. Together, these results suggest that when given a choice, LE rats prefer to play with their own strain, but when they do not have a choice, any strain will do. Given that the testing paradigm can greatly influence the results obtained and the conclusions drawn, our findings highlight the need to consider the research question(s) being asked when determining the most appropriate paradigms to employ.

## Introduction

Though there are many forms of play behavior (Burghardt, 2005; Fagen, 1981; Pellis and Pellis, 2009), rough-and-tumble play (RTP) or play fighting is the most common form of social play reported and studied, especially in young mammals (Pellis et al., 2024; Pellis and Pellis, 1998). Regardless of the species, RTP is centered around animals competing for an advantage—such as access to a particular location on the partner’s body—while also showing some degree of cooperation, making the interactions somewhat reciprocal (Pellis et al., 2024). Laboratory rats have been the most intensively studied species and have provided many of the major insights into the neurobiology and developmental consequences of RTP (Achterberg and Vanderschuren, 2023; Pellis et al., 2023).

Rats engage in RTP both as juveniles and adults (Pellis and Pellis, 1990; Thor and Holloway, 1984b), although its occurrence peaks during the juvenile period, between 30 and 40 days after birth (Panksepp, 1981; Pellis and Pellis, 1990; Thor and Holloway, 1984a). Juvenile male rats are frequently reported to engage in RTP more than females (e.g., Meaney, 1988; Meaney and Stewart, 1981; Thor and Holloway, 1983), however, this is not always the case (Northcutt and Nwankwo, 2018). Indeed, it seems that other factors, particularly how the rats are reared as juveniles can greatly modify the prominence of this sex difference. For example, when reared in multi-animal, mixed-sex groups, males play more than females. However, when rats are reared with one, same-sex partner, sex differences in RTP are diminished (Himmler et al., 2016a). Regardless of sex differences in the frequency of RTP, in rats of both sexes RTP involves attack and defense of the nape of the neck, which is gently nuzzled with the snout if contacted (Pellis and Pellis, 1987; Siviy and Panksepp, 1987). Typically, for 80% or more of nape attacks the recipient uses defensive tactics to prevent contact, such as rolling to supine so that its nape is pressed to the ground and cannot be contacted (Himmler et al., 2013a; Pellis et al., 2022a). While sex differences emerge in how rats play as they age (Pellis, 2002), during the juvenile period, males and females adopt similar defensive tactics (Pellis and Pellis, 1990). Though most intensely studied in domesticated strains of rat in the laboratory (e.g., Achterberg et al., 2014, 2019; Himmler et al., 2014b; Siviy et al., 2017; Veenema et al., 2013), this pattern of attack and defense of the nape is also observed in wild populations (Calhoun, 1963) and wild-caught rats observed under laboratory conditions (Barnett, 1958; Himmler et al., 2013b).

Given that in rats RTP has been shown to be a naturally motivated behavior (Siviy, 2016; Vanderschuren, 2010), does not require training (Pellis et al., 2022b), and has a considerable degree of individual variation (Achterberg et al., 2023; Ham and Pellis, 2023; Lesscher et al., 2021), this behavior has become increasingly used as means to study social behavior and its development. For example, play has been implicated in the development of executive functions, social skills, and cognitive flexibility, with anatomical and physiological changes of the areas of the prefrontal cortex associated with these capabilities (Bijlsma et al., 2022, 2024; Ham et al., 2024; Schneider et al., 2016; Stark and Pellis, 2020, 2021).

Though every strain of laboratory rat studied to date targets the nape of the neck during RTP (Himmler et al., 2016b; Himmler et al., 2014a; Himmler et al., 2014b), there is variation in how much they play. For example, Sprague Dawley rats (SD) rats typically initiate around 1.5 times more play than Long Evans (LE) rats (Himmler et al., 2014b). Fischer 344 (F344) rats typically launch half as many attacks as LEs when tested with partners from other strains (Siviy et al., 1997, 2003; Stark et al., 2021), but more than LE rats when tested in same-strain pairs (Ham et al., 2024). As well as the variation in play frequency both within and among strains, how the rats play also varies from strain to strain. For example, compared to LE rats, SD and F344 rats are more likely to evade a playful attack, by swerving or running away, than rolling to supine (Ham et al., 2024; Himmler et al., 2014b; Orsucci et al., 2024; Siviy et al., 2023).

Given the differences in playfulness and defensive tactics, some combinations of strains are more compatible than others. For example, LE, SD, and F344 rats tend to retain their strain-typical preferences in tactics of defense when tested with unfamiliar partners from other strains (Himmler et al., 2014a; Schneider et al., 2016; Siviy et al., 1997; Siviy et al., 2003; Siviy et al., 2011; Siviy et al., 2017; Siviy et al., 2023; Stark et al., 2021). If reared together, some strains, such as LE and SD, tend to modify their play styles to more closely match the style of the other strain (Himmler et al., 2014a), but others, such as F344 rats, maintain their strain-typical style regardless of the strain with which they are reared (Schneider et al., 2016; Siviy et al., 2017; Stark et al., 2021). Critically, F344 rats, unlike SD and LE rats, seem unable to create the opportunities for role reversals and symmetry in playful attacks when playing with a partner from a different strain (Ham et al., 2024; Schneider et al., 2016; Stark et al., 2021), and so ensure the experiences of reciprocity that are essential for play to provide its developmental benefits (Pellis et al., 2023). Because of these strain differences, given the choice among potential play partners, we predicted that LE rats should prefer their own strain over SD and F344 rats, but likely prefer SD over F344. Partner preferences among adults in non-playful contexts support this prediction. LE and SD rats prefer to socialize with their own strain or even each other, before socializing with a F344 partner (Kiyokawa et al., 2022; Kogo et al., 2021).

Typically, when testing play in rats, the “dyadic paradigm” is used, where two rats are placed in a neutral arena after some period of social isolation (Achterberg and Vanderschuren, 2023; Pellis et al., 2022a). A limitation with this paradigm is that rats are forced to play with partners selected by the experimenter, and play ensues even if the partner is not optimal (Achterberg et al., 2023). Indeed, if given the choice of multiple partners, in a group testing paradigm, rats will tend to preferentially initiate more play with some members of the group than with others (Ham and Pellis, 2023, 2024, 2025). Such a group play paradigm more closely resembles the situation in which rats evolved, where they not only have multiple littermates as potential partners, but also peers from the litters of other females in the colony (Meaney and Stewart, 1981; Pellis and Pellis, 1997; Schweinfurth, 2020). Therefore, to test our prediction, we used a group paradigm to assess play preferences in partner selection across strains.

Groups of four rats, comprising two LE rats and one each of SD and F344 rats, were tested. One LE rat was selected as the focal rat and the frequency of nape attacks it initiated toward the other three rats was used as a means to determine partner preference. Because of the quantitative (number of attacks) and qualitative (preferred defense tactics) differences among these strains discussed above, our prediction was that the order of preference would be LE > SD > F344. The focal LE rats were also tested in dyads with the three different strains, and we predicted that LE rats would play with LE, SD, and F344 rats at similar frequencies as they satisfy their motivation to play with the only partner available (Ham et al., 2024; Himmler et al., 2014a; Stark et al., 2021). Finally, we used socially conditioned place preference (sCPP) (Achterberg et al., 2012, 2014; Trezza et al., 2009, 2011), and predicted that the focal LE rats would prefer to spend time in a context in which they had played with a same-strain partner over one in which they had played with a less compatible F344 partner.

To assess the LE rats’ preference in the sCPP test further, their ultrasonic vocalizations were recorded. As 50 kHz calls are emitted in positively affective contexts, including play (Burgdorf et al., 2008), and their rate of emission is increased in a play anticipation paradigm (Burke et al., 2017, 2021; Knutson et al., 1998) but reduced during dyadic play encounters between LE and F344 partners (Stark et al., 2021), we predicted that more calls would be emitted when accessing the preferred box in the sCPP trials.

## Materials and methods

### Ethics

All care and testing procedures were approved by the University of Lethbridge Animal Welfare Committee (Protocol #2408) in compliance with guidelines from the Canadian Council for Animal Care.

### Subjects

Sixteen LE, eight SD, and eight F344 male rats were purchased from Charles River Laboratories (Kingston, New York) and arrived at the Canadian Center for Behavioral Neuroscience at 24 days of age. Upon arrival, the rats were housed in same-strain pairs. Animals were housed in double decker cages with corncob bedding. Two days after arriving, the animals were handled and weighed daily by the experimenters to habituate the rats to the experimenters, being handled, and being weighed. Food and water provided were *ad libitum*. The rats were housed on a 12-h light-dark cycle (lights on between 7:30 a.m. and 7:30 p.m.) in a room maintained at a constant temperature of 21–23°C.

### Apparatus

#### Group play

Play in groups was tested in a clear Plexiglas enclosure (80 × 80 × 50 cm). The floor of the enclosure was covered with corncob bedding. Interactions were filmed with a digital video camera (Sony Handycam FDR-AX53) which was placed above the enclosure at a 90° angle so that all four rats could be visualized simultaneously. Red lights were used to illuminate the enclosure.

#### Dyadic play

Play in pairs was tested in a clear Plexiglas enclosure (50 × 50 × 50 cm). The floor of the enclosure was covered with corncob bedding. Interactions were filmed with a digital video camera which was place outside of the enclosure obliquely, at a 45° angle. Red lights were used to illuminate the enclosure.

#### Social conditioned place preference

**FIGURE 1:**
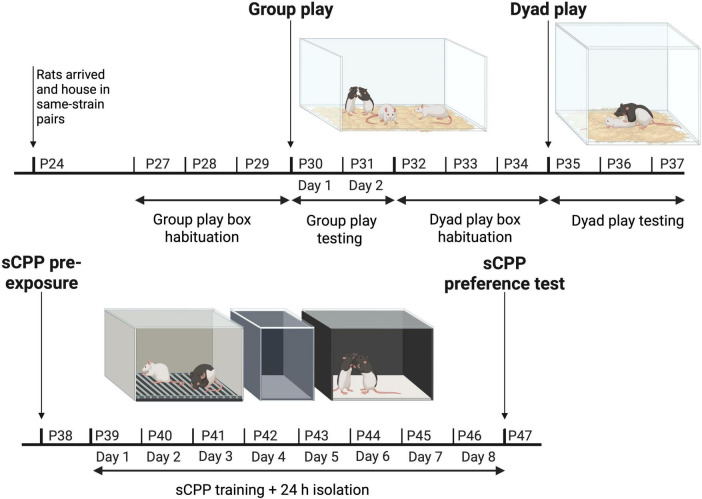
The experimental paradigm illustrating how the animals were tested in groups on postnatal day (P) P30 and P31, tested in pairs between P35–37, and pre-exposed, trained, and tested using social conditioned place preference (sCPP) on P38, P39–P46, and P47, respectively. Created with BioRender.com.

sCPP was tested in an apparatus comprised of three compartments, each with a removable Plexiglas lid. Two of the compartments were large and equal in size (41 × 41 × 50 cm) separated by a smaller alleyway (16.5 × 11 × 11 cm). The two large enclosures, where the animals were conditioned to a stimulus, had different visual and tactile cues (Figure 1). One of the contexts was a white box with a black floor, with stainless-steel bars spaced 1.5 cm apart lining the floor. The other was a black box with a white floor that did not have bars along the floor. Both chambers had Pettersson M500-384 USD ultrasound microphones (Pettersson Elektronik AB, Sweden) affixed to the Plexiglas lids so that USV could be recorded. Sessions were filmed with a digital camera which was placed above the enclosures at about a 70° angle.

### Procedure

Eight of the 16 LE rats ordered were designated as “focal” rats and were the primary subjects of this study. These eight focal rats were housed together in pairs, and tested and scored in both the group and dyadic contexts for play, and finally, they were tested in the sCPP paradigm (Figure 1).

#### Group play

At 27 days of age, the rats of each strain were habituated to the test enclosure for 20 min, with their cage mates, in red light. This was done for 3 consecutive days between 7:30 a.m. and 3:00 p.m. At 30 days of age, group play testing began. Before testing, rats were weighed, their fur marked with a permanent marker pen (Sharpie^®^), and socially isolated for 2.5 h to increase their playfulness (Pellis et al., 2022a). Food and water were provided *ad libitum* during social isolation. Groups consisting of two LE (one being the focal LE), one SD, and one F344, all of which were unfamiliar with one another, were placed into the test enclosure, in red light, for 20 min and filmed. The group play trials were repeated for 2 days, switching the individuals within each group so that they were again all unfamiliar on the second test day. Following a test session, the animals were placed back into their respective home cages.

#### Dyadic play

As the dyadic enclosure was located in a different room and is a different size, at 32 days of age, the rats were habituated to the dyadic test enclosure for 10 min, with their cage mates, in red light. This was done for 3 consecutive days between 7:30 a.m. and 3:00 p.m. At 35 days of age, dyad play testing began. Before testing, rats were weighed, marked with a permanent marker pen (Sharpie^®^), and socially isolated for 2.5 h. Again, food and water were provided *ad libitum* during social isolation. Pairs of either two LEs, one LE and one SD, or one LE and one F344, who were unfamiliar with one another, were placed into the test enclosure, in red light, for 10 min and filmed. The dyadic play trials were run over 3 days, counterbalancing the strains paired with the focal rats. Following all test sessions, the animals were placed back into their respective home cages.

#### Socially conditioned place preference

##### Pre-exposure

Using a modified version of sCPP (see Achterberg et al., 2012, 2014; Trezza et al., 2009, 2011), at 38 days of age, the rats were placed into the middle alley chamber, which was connected to both large boxes, one at a time. Under red-light conditions, the rats were allowed to explore both chambers freely for 15 min while being video recorded. Time spent in both chambers, and the middle alley, was measured. According to the pre-exposure results, the rats were counterbalanced such that any innate preferences for either context was balanced for partner × context pairing. Following pre-exposure, all rats, including the partner rats, were socially isolated with food and water provided *ad libitum*.

##### Training

Training began approximately 24 h after pre-exposure. The two chambers were sealed, making the middle alleyway and the opposing chamber inaccessible. Half the rats experienced an LE partner in the white box while the other half experienced an LE partner in the black box. In the opposite context, they were paired with a F344. On days 1, 3, 5, and 7 of the training, rats were placed in one of the chambers for 15 min with an unfamiliar partner (either LE or F344). In the afternoon, they were placed in the opposite box, for 15 min, with the opposite partner. On days 2, 4, 6, and 8 of training, the order of the two daily sessions was inverted. For example, on day 1, focal Rat A would be partnered in the morning with an LE rat in the white box. In the afternoon, they would be partnered a F344 rat in the black box. On day 2, focal Rat A would be partner with a F344 rat in the black box in the morning, and an LE in the white box in the afternoon. Morning and afternoon sessions, on any given day, were separated by at least 4 h. Partners were counterbalanced so that half of the partners experienced an LE in the morning and half experienced a F344 in the morning. Similarly, the focal rats starting a training session was counterbalanced so that if Rat A started first on day 1, he would start last on Day 2. Partners were rotated each day so that they were always unfamiliar with the focal rat to preclude the formation of partner preferences and ensure that any preferences formed were for the strain and not the particular individual with which they were interacting. Training was done for 8 consecutive days. Following morning and afternoon training session, the rats were placed back into social isolation.

##### Preference test

Twenty-four hours after the 8th and final day of training, the preference test was conducted (Figure 1). Both boxes were unsealed, and the gray alleyway was replaced so that the two boxes were connected. Like pre-exposure, rats were placed in the middle alleyway and allowed to explore both boxes freely for 15 min under red-light conditions. The test sessions were filmed, and the recordings were subsequently used to score the time spent in the two boxes and the alleyway.

### Behavioral analyses

#### Group play

Following group play trials, the videos were analyzed using a combination of normal speed and frame-by-frame analysis to score play behavior (Himmler et al., 2013a; Pellis et al., 2022a). As we were interested in how a focal rat would distribute its play among the potential play partners, we used a focal follow approach (Bateson and Martin, 2021) and scored the distribution of nape attacks directed toward each partner by the focal LE rat. Playful attacks were scored when the snout of one rat was in contact with, or directed toward, the nape of another rat (Pellis et al., 2022a). The frequency of play, and latency to first and last attack, were scored for both days.

#### Dyadic play

We measured playful RTP bouts to determine if the focal LE rats played with one strain more than the others. Like above, a playful attack was scored when the snout of one rat was in contact, or directed toward, the nape of another rat (Pellis et al., 2022a).

#### Socially conditioned place preference

To calculate if there was a preference for a particular chamber during the pre-test, the time spent in each of the three sections of the apparatus, was calculated. Time in a box was accumulated when both forepaws and half the body were past the threshold of the doorway into one of the boxes (McDonald et al., 2023). Similarly, time spent in each box was scored by an observer blind to the training conditions after the preference test to determine if a certain box was preferred.

Based on previous studies, we assumed that LE-F344 pairs would have less reciprocal play relationships than LE-LE pairs; thus, we expected an LE partner would be more compatible than a F344 partner despite the absolute frequency of play being similar (Ham et al., 2024; Stark et al., 2021). However, to be certain that the play experiences differed when paired with a F344 vs. a LE during training, the encounters in both the morning and afternoon on training days 1, 5, and 8 (beginning, middle, and end of the training, respectively) were scored and averaged. The number of attacks launched by the focal rat were scored. Additionally, the amount of play the partner initiated was scored so we could quantify how playful the partner rats were, and thus, how rewarding they were as a play partner (Lampe et al., 2019). If bedding is not provided in the play enclosure, rats switch defense strategies, reducing the number of rotations to supine (Pellis and Pellis, 2021). As we used two different floors, we scored the number of attacks resulting in a pin to determine if the floor biased how rats played.

#### Ultrasonic vocalizations

The vocal recordings from the preference tests were analyzed using Raven Pro 1.6 software (Bioacoustics Research Program, Cornell Lab of Ornithology, Ithaca, NY). Raven Pro generated spectrograms with a 256-sample Hann window. Using this window, vocalizations were manually selected and labeled by a scorer blind to the play partner context pairing. Each of the USV was scored according to the 14 category schema provided by Wright et al. (2010). These data were used to compare the number and type of calls made in each context. The total number of vocalizations and call types were compared. In addition, the percentage of total calls, and each call type, was calculated to determine if more calls or more calls of a certain type were made in either context. Finally, the rate at which calls were made was calculated (number of calls/total time spent in the context).

### Statistical analyses

All plots were created using Prism version 10 (GraphPad Software).

#### Group play

The frequency of nape attacks and latency to first and last attack of each partner, on each day, were compared using two-way repeated measures ANOVAs with Bonferroni corrections.

#### Dyadic play

The frequency of nape attacks initiated by each pair in all strain combinations, as well as the frequency of nape attacks initiated by the focal rat in each of those pairings was compared using Freidman’s tests in Prism. After plotting the data, we noted a large degree of variation in the latency to first attack on Day 1, and so we performed a robust regression and outlier removal (ROUT) test, with a false discovery rate (Q) set to 1% using Prism. However, no outliers were detected.

#### Socially conditioned place preference

Using Prism, paired *t-*tests were used to determine if the rats had an inherent bias for either the black or white box during the pre-test. After conditioning, we used a paired *t-*test to test if the rats formed a preference for the LE or F344 context, as well as for either the black or white box. The frequency of play behavior initiated by the focal rat and how they defended themselves from playful attacks by the LE and F344 partners, and whether this differed in the black and white boxes, was tested with paired *t-*tests. We used unpaired *t-*tests to determine if the frequency of play behavior initiated by the partner rats in either the LE or F344 context and the black or white box was significantly different. This was repeated to determine if the partner rats defended themselves differently from playful attacks by different partners or different boxes.

##### Vocalizations

Two-way repeated measures ANOVAs were used to compare the number, distribution, and rate of vocalizations in the contexts with different partners and different boxes.

## Results

### Group play

**FIGURE 2:**
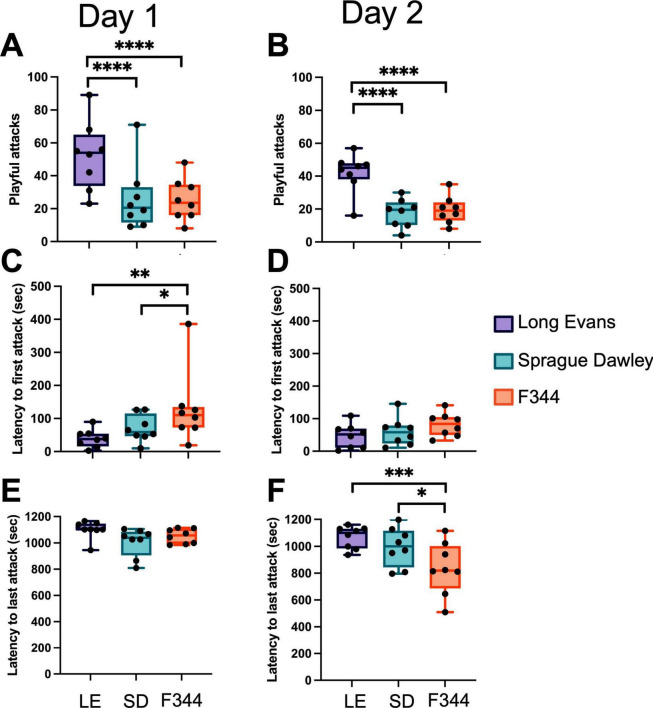
On both day 1 **(A)** and day 2 **(B)** of group play, the frequency of nape attacks initiated by the focal rat and directed toward a LE, SD, and F344 varied with focal rats directing more of their play toward LE partners than SD and F344s. LE partners were attacked faster than SD and F344 partners on day 1 **(C)** but not day 2 **(D)**. No difference was found in latency to last attack on day 1 **(E)**, but on day 2 **(F)**, LE rats were attacked later in the session than both SD and F344s. *p < 0.05; **p < 0.01; ***p < 0.001; ****p < 0.0001.

We found that the focal rats did not play with each strain equally [*F*(2, 14) = 64.18, *p* < 0.0001] (Figure 2A), however, there was no effect of day [*F*(1, 7) = 2.40, *p* = 0.166] or strain × day [*F*(2, 14) = 0.70, *p* = 0.515]. *Post-hoc* tests showed that focal rats played with LE partners more than SD (*p* < 0.0001) and F344 (*p* < 0.0001) partners on Day 1. Similarly, on Day 2 (Figure 2B), focal rats played more with LE partners than SD (*p* < 0.0001) and F344 (*p* < 0.0001) partners.

When the latency to first attack was compared, we found that there was a significant effect of strain [*F*(2, 14) = 6.815, *p* = 0.009], but not day [*F*(1, 7) = 0.77, *p* = 0.411] or strain × day [*F*(2, 14) = 1.968, *p* = 0.177]. *Post-hoc* tests showed that there was no difference in time to first attack between LE vs. SD, but F344 partners were the last to be attacked compared to LE and SD (*p* = 0.002; *p* = 0.03, respectively) on Day 1 (Figure 2C). No differences were found in latency to first attack on Day 2 (Figure 2D). When the latency to last attack among partners was compared, we found a significant effect of strain [*F*(2, 14) = 5.07, *p* = 0.022], day [*F*(1, 7) = 6.38, *p* = 0.040], and strain × day [*F*(2, 14) = 6.21, *p* = 0.012]. *Post-hoc* tests showed that there was no difference in latency to last attack between strains on Day 1 (Figure 2E). However, on Day 2 (Figure 2F), LE and SD were attacked later in the test session than the F344 rats (*p* = 0.0005; *p* = 0.010, respectively). *Post-hoc* tests also revealed that F344 rats were attacked sooner in the test session on Day 1 compared to Day 2 (*p* = 0.0003).

### Dyadic play

**FIGURE 3:**
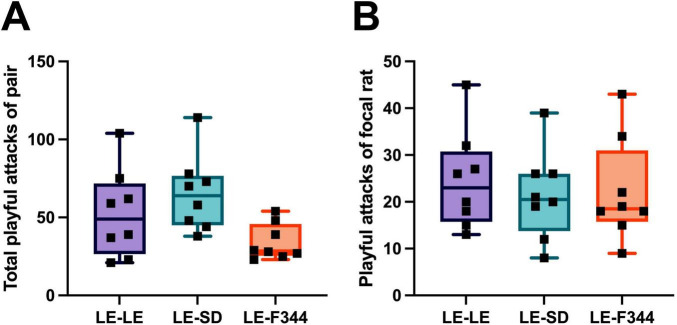
When the frequency of attacks by both partners in the pair was summed for dyadic play, no difference was found between LE-LE, LE-SD, nor LE-F344 pairs **(A)**. Similar, the frequency of play initiated by the focal rat when playing with either an LE, SD, or F344 partner did not differ **(B)**.

The total play between pair mates did not differ significantly in the any of the strain combinations (Figure 3A) [*X*^2^(2) = 5.87, *p* = 0.053], although there was a trend for less play in the pairs with F344 partners. Similarly, LE focal rats launched a comparable number of nape attacks (Figure 3B), regardless of the strain with which they were partnered [*X*^2^(2) = 1.75, *p* = 0.531].

### Socially conditioned place preference

**FIGURE 4:**
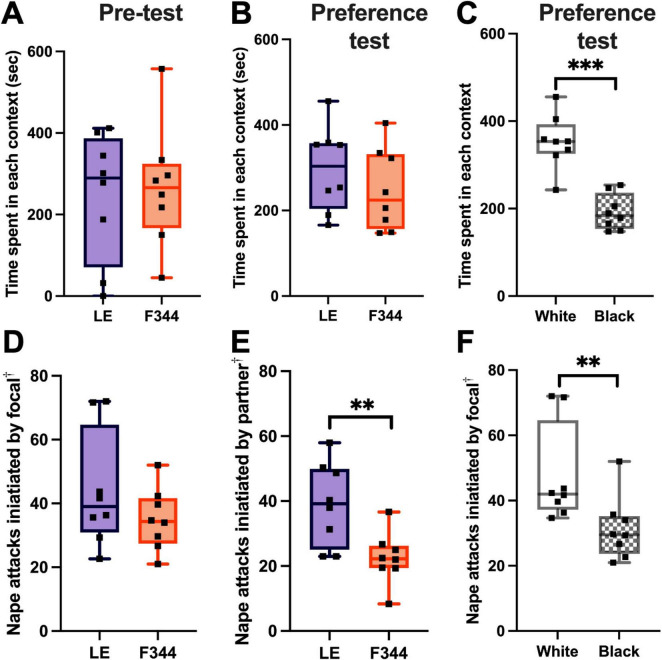
The time spent in each box was not different between the to-be-paired LE and to-be-paired F344 contexts **(A)**. After 8 days of condition place preference training, focal rats did not prefer the LE social context over the F344 context **(B)**. The focal rats did, however, prefer the white box over the black box, regardless of the social partner they experienced in the box **(C)**. The focal rats did not initiate more play with LE or F344 rats **(D)**. However, LE partners played more than F344 partners **(E)**. Though focal rats did not play more depending on the social context, the focal rats initiated more play in the white box compared to the black box **(F)**. ^†^ Attacks were measured on three different days (day 1, 5, and 8) and the average frequency of attacks across the 3 days was used. ^**^p < 0.01; ^***^p < 0.001.

During the pre-test, we found that there was no bias for the box that was to-be-paired with an LE nor the box that was to-be-paired with a F344 [*t*(7) = 0.216, *p* = 0.835] (Figure 4A). After 8 days of training, we found that the focal rats did not form a preference for either the LE or F344 paired contexts [*t*(7) = 0.773, *p* = 0.465] (Figure 4B). However, when further analyzing the data, we found that the rats significantly preferred the white box during the preference test [*t*(7) = 6.261, *p* = 0.0004], regardless of whether they were partnered with a LE or a F344 in the white box (Figure 4C).

After scoring the interactions on training Days 1, 5, and 8, we found that the focal rats did not play with LE partners more than F344 partners across the three sample days [*t*(7) = 1.431, *p* = 0.196] (Figure 4D). The focal rats, however, did defend attacks by rolling over into a supine position when playing with an LE partner (mean number of supine defenses: 6.29) more than when with a F344 partner (mean number of supine defenses: 1.17) [*t*(7) = 0.230, *p* = 0.0131]. LE partners, on average, initiated more play than the F344 partners [*t*(14) = 3.102, *p* = 0.0078] (Figure 4E), but did not defend themselves with more supine defenses when playing [*t*(14) = 1.772, *p* = 0.098].

Given that focal LE rats preferred the white box over the black box, we compared whether the focal rats initiated more play in the white box. We found that LE rats launched more playful attacks in the white than the black box, across the 3 days measured [*t*(7) = 4.336, *p* = 0.0034] (Figure 4F). The focal rats did not, however, defend themselves by rolling to a supine position more in either the white or black box [*t*(7) = 0.865, *p* = 0.416]. No difference was found in the frequency of play initiated by the partner in either the white or black box [*t*(14) = 0.652, *p* = 0.525] or in the number of supine defenses they used [*t*(14) = 1.104, *p* = 0.288].

**FIGURE 5:**
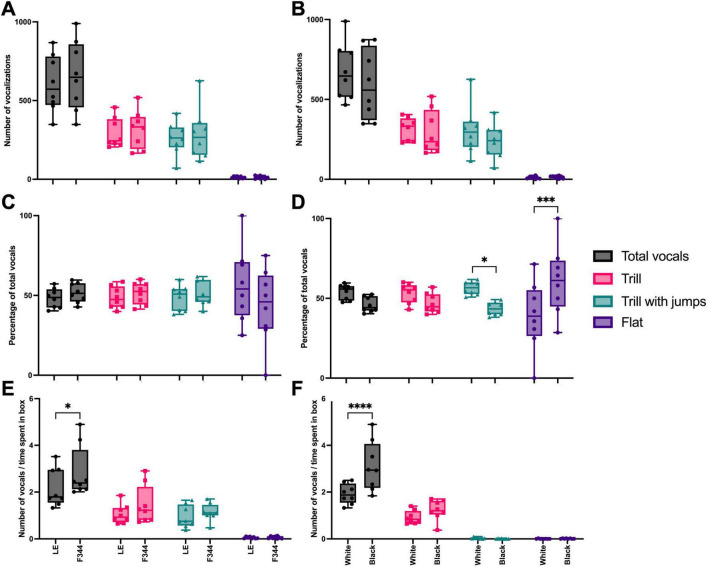
The total number of vocalizations, trills, trill with jumps, and flat calls emitted in each paired context (i.e., LE or F344) did not significantly differ **(A)**. Similarly, the number of calls did not differ depending on the box (i.e., white or black) **(B)**. When calculated as a percentage, the total number of calls and the types of calls emitted did not differ between paired contexts **(C)**. However, focal rats did emit more trill with jumps in the white box, compared to the black box, and more flat calls in the black box, compared to the white box **(D)**. When calculated as a rate, more calls were made in the F344 context than the LE context **(E)** and more calls were made in the black box than the white **(F)**, however, the call type did not differ between either context or box. *p < 0.05; ***p < 0.001; ****p < 0.0001.

Of all the USV emitted, the majority (93%) were trills, trill with jumps, and flat calls, so these were the call types we analyzed further. There was no interaction effect between the number of ultrasonic vocalizations recorded and the context paired with either strain [*F*(3, 42) = 0.165, *p* = 0.919] (Figure 5A) nor for the white or black boxes [*F*(3, 42) = 0.622, *p* = 0.605] during the preference test (Figure 5B). When we compared the percentage of vocalizations emitted in the LE vs. F344 contexts, no interaction effect was found in the distribution of calls [*F*(3, 42) = 1.524, *p* = 0.222] (Figure 5C). However, an interaction effect was found between the color of the box and the type of vocalizations emitted [*F*(3, 42) = 8.450, *p* = 0.0002], with the focal rats emitting more trill with jumps in the white box and more flat calls in the black box (Figure 5D). Given that the focal rats did not spend equal amounts of time in each chamber, we also assessed the rate at which each vocalization type was used in each context. Focal rats vocalized at a different rate depending on the context [*F*(3, 42) = 72.85, *p* < 0.0001], vocalizing at a higher rate in the F344 paired context (Figure 5E). Similarly, focal rats vocalized at a different rate depending on whether they were in the white or black box [*F*(3, 42) = 7.777, *p* = 0.0003], emitting vocalizations at a higher rate in the black context (Figure 5F). However, for neither partner pairing nor box type pairing were there any significant differences in call types emitted.

## Discussion

Given that there is a considerable degree of variation in both the amount of play and the style of play among strains of rats (Himmler et al., 2014b; Siviy et al., 2023), we aimed to determine whether rats have play partner preferences based on strain identity in different contexts. To do so, we tested juvenile male LE rats and in three conditions. First, we tested the rats with unfamiliar partners in a group setting where they had the choice to play with either an LE, SD, or F344 rat. In the group play test, which was repeated twice on 2 consecutive days, focal rats consistently played more with the same-strain LE partner over the SD and F344. Unexpectedly though, SD were not preferred over F344 rats. However, both LE and SD partners were playfully attacked before the F344 rats were on Day 1 of testing, suggesting some preference of SD rats over F344 rats. Following the group play testing, the focal rats were paired with each strain, one at a time, in a dyadic play encounter. We found that the focal rats directed the same amount of play to all partners, suggesting that when rats do not have a choice, they will play with partners of different strains equally. That is, despite there being considerable differences in both the amount and the style of RTP among strains of rats (Himmler et al., 2014b; Siviy et al., 2023), when playing with members of a different strain in a dyadic context, the strain-typical differences are reduced, leading to a convergence in both frequency and style of play (Ham et al., 2024; Reinhart et al., 2006; Siviy et al., 1997, 2003; Stark et al., 2021). Nevertheless, it should also be noted that prior rearing with a different strain can affect the degree of such convergence (Himmler et al., 2014a). That is, subject to modifying influences of the test paradigm used (see also Argue and McCarthy, 2015b), the dyadic test context can lead to the partners accommodating to each others’ play styles, but again, some strains, such as the F344, are more resistant to such accommodation than others (Ham et al., 2024; Siviy et al., 1997, 2003, 2017). When restricted to playing with a particular partner in the dyadic test paradigm, rats seem to adjust their play (Achterberg et al., 2023) in a manner that may not be necessary when multiple partners are available (Ham and Pellis, 2023, 2024, 2025), and, as shown here, a partner from a less suitable strain, can be avoided in preference to members of more suitable strains. Finally, we used a modified version of the play-induced socially conditioned place preference paradigm (Achterberg et al., 2014; Trezza et al., 2009, 2011) to determine if the focal LE rats would prefer the context in which they played with a compatible, same-strain play partner over an incompatible, F344 partner (Ham et al., 2024; Stark et al., 2021). The focal rats preferred the white box over the black box, and this was the box in which they played more, irrespective of the strain of the play partner. Interestingly, the focal rats vocalized at a higher rate in the black box, and in boxes that had been rewarded with F344 partners, indicating that while some parameters of 50 kHz, such as the number and types of calls may reflect positive affective states (Burke et al., 2017, 2021; Knutson et al., 1998), others, like rate of emission, may reflect overall level of arousal (e.g., Gaub et al., 2016; Raza et al., 2015). Consequently, it would seem that, at some level, the rats are distinguishing between both the box and the strain of the training partner.

### Play is better than no play

When given a choice, we found that focal rats significantly prefer their own strain, however, when the choice in play partner is removed, the rats play equally with all partners (F344 or SD). That is, in a dyadic set-up, as is done in most play research, the focal rat will play with a less preferred partner as much as with a preferred partner. This may be in part due to the rats having been socially isolated (even if just for 2.5 h) before testing play. Though briefly isolating the rats to ensure they are motivated to play before testing is a commonly used technique (Himmler et al., 2013a; Pellis et al., 2022a), this increased motivation to play might influence the results from the dyadic tests. For instance, if the rats are highly motivated to play, and the only partner they have is a suboptimal one, the motivation to play may outweigh the reluctance to play with such a partner. Perhaps, if they were not isolated at all, and so not as motivated to play, they would only play with the LE partners and avoid both the SD and F344 partners during the group test. How the duration of social isolation and motivation to play affects partner selection will be the focus of future studies; however, preliminary results from our laboratory indicate that LE focal rats are not less selective with increased isolation time suggesting that the time spent in isolation did not bias the strength of their partner preferences. Alternatively, the lack of discrimination between SD and F344 rats in the group test may be due to the presence of an LE rat. As in the non-play social tests using adults, when given the choice, LE rats preferred to interact with other LE rats over other strains, but when the only choice available were SD rats, they preferred the SD rats over the F344 rats (Kiyokawa et al., 2022; Kogo et al., 2021). Similarly, if a group test for play only involved a choice between an SD and a F344 rat, LE rats may show a preference for SD play partners. That is, cross-strain preferences may still exist, but to characterize them, the appropriate testing paradigm needs to be identified.

That context is critical for identifying the occurrence of behaviors is one implication from this study, as LE rats tested in pairs did not show the same play partner preferences as when tested in groups. It should be noted that we chose F344 and SD rats because their style of play differs markedly from that of LE rats (Himmler et al., 2014a; Himmler et al., 2014b; Siviy et al., 1997; Stark et al., 2021), which may possibly exaggerate the effects of partner preference in mixed-strain group play. Indeed, we selected the three strains we did as they each play differently. However, their differences are graded. SD share a more similar play style to LE than F344 (Himmler et al., 2016b; Himmler et al., 2014a; Himmler et al., 2014b). Had we used strains that play more similarly to LE rats, such as Brown Norway and Wistar rats (Himmler et al., 2014b), we may not have observed such a strong preference during the group play testing. Still, even subtle differences within a strain appear to be sufficient for rats to form partner preferences in a group setting. For example, LE rats playing in groups of same-strain, same-sex partners, form partner preferences whether playing with strangers (Ham and Pellis, 2024), cage mates (Ham and Pellis, 2025), or with partners of mixed familiarity (Ham and Pellis, 2023). Nonetheless, as stated above, when rats do not have a choice and are motivated to play, they will play with whatever partner is available. However, when provided the opportunity to choose, they will select a same-strain partner (present study), or a same-strain partner with particular characteristics (Ham and Pellis, 2023, 2024, 2025). These contradictory results highlight the need to study ethologically relevant behaviors and to test animals under more naturalistic conditions (d’Isa and Gerlai, 2023; Dennis et al., 2021; Kondrakiewicz et al., 2019) as, depending on the question being tested, the paradigm used could bias the results. Indeed, had we only tested the rats using the dyadic paradigm, we may have concluded that LE rats do not prefer their own strain. For general or fundamental phenomena, such as the effect of pharmacological manipulation and deciphering which brain regions are involved in play, using play frequency, duration, and the microstructure of play as tested under the dyadic test set-up is sufficient for now (Achterberg et al., 2016, 2019; Achterberg and Vanderschuren, 2020; Trezza and Vanderschuren, 2008; VanRyzin et al., 2019; Veenema et al., 2013). However, to answer more ethologically relevant questions, such as partner preference due to experimental manipulations or treatments, the group set-up may be better suited (Ham and Pellis, 2023, 2024; Holman et al., 2019).

In addition to partner discrimination, the focal LE rats played far more when in a group setting than when in a dyadic setting. On day 1, the focal rats launched on average 102.38 attacks, and 78.88 on day 2, that is roughly 4.2 times and 3.3 times more play than when tested in the dyadic context (Figure 3B). This large difference between test conditions suggests that there may be a group contagion effect whereby rats are more motivated to play when more individuals are present. Alternatively, it could be that the novelty of choice is motivating given that the animals were pair housed and so were never offered the opportunity to choose a partner. In either case, the increased frequency of play when in groups cannot be explained by the natural increase and decrease in playfulness with age. Play in rats is most frequent between 30 and 40 days of age, typically peaking around 35–36 days of age (Panksepp, 1981; Pellis and Pellis, 1990; Thor and Holloway, 1984a). In the present study, group testing occurred when the rats were 30 and 31 days old, and dyadic testing around 35 days, so, if anything, play should have been more frequent when tested in pairs.

### Socially conditioned place preference: partner or context?

Previous studies have shown that isolated rats prefer contexts that are paired with RTP over contexts without the opportunity to play (Achterberg et al., 2012, 2014; Trezza et al., 2009, 2011). Here, a slightly altered methodology was used to determine if rats would prefer a context in which they encounter a playful same-strain partner over a context where they encounter a playful partner from a different strain. We predicted that rats would prefer the context in which they had a same-strain partner because the play experiences would be more compatible, both in style and frequency.

To our surprise, we found that the focal LE rats did not prefer the context that was paired with a same-strain partner. Instead, they preferred a particular box, regardless of the strain of the partner. The focal rats in our study significantly preferred the white box. Following previous protocols (Achterberg et al., 2012, 2014; Trezza et al., 2009, 2011), we varied the color of the enclosure and the texture of the floor so the rats could easily distinguish between the two contexts. However, unlike the previous sCPP studies where fine metal mesh and wide metal mesh flooring was used to distinguish the two contexts, we had one floor with metal rods lining the surface while the other floor was smooth. Despite us wanting the floor texture varied so to provide the rats an additional cue, we may have inadvertently biased the play experiences.

Depending on the context or the enclosure in which rats are tested, both the frequency and structure of play can be altered. For example, when tested in circular arenas, rats play significantly less than when tested in square or rectangular ones (Hole and Einon, 1984). If no bedding is provided in the enclosure, the rats will switch defense strategies, favoring evasive strategies over rotating to a supine position (Pellis and Pellis, 2021). Given that RTP in rats can be rough, it is suspected that having bedding to cushion the wrestling ensures that the play remains comfortable (Pellis and Pellis, 2021). Knowing this, we selected chambers that were both square (and of the exact same dimensions) and devoid of bedding, so as not to have one with bedding and one without. We believe this was achieved as we did not find a difference in the number of supine defenses performed between boxes.

In addition to the texture of the floor, the color of the walls and floors of the boxes were varied and this could have possibly biased the animals even though it is standard to both alter the floor and wall color (e.g., Martínez et al., 1995; McDonald et al., 2021; Roux et al., 2002). In the white enclosure, the floors were black while in the black enclosure the floors were white. It remains unclear if the color of the walls and/or floors, texture of the floor, or both biased the rats’ behavior. For instance, considering the box preference that we observed, it is possible that a black floor is less alerting to rats and hence favors play. In future experiments, these factors (wall color, floor color, and floor texture) should be separated and tested singularly for their effects on RTP.

Regardless of the environmental cues that were salient to the rats, they played less in the black box during the training days and emitted different vocalizations during the preference tests depending on the context. In the white box, rats emitted more trills with jumps than in the black box. These trills have been associated with positive experiences (Burke et al., 2022; Reinhold et al., 2019) and seem to be used in coordinating play behavior (Burke et al., 2018, 2020; Kisko et al., 2015). Conversely, 50 kHz flat calls, which are often used as contact calls (Brudzynski, 2015), frequently emitted after having been socially isolated (Wöhr et al., 2008), were emitted more in the black, unpreferred box.

Given that the animals did prefer a box, and they preferred the box they played in more, it seems that like previous studies (Achterberg et al., 2012, 2014; Trezza et al., 2009, 2011), the rats in this study formed preferences based on their play experiences. This preference, though, was not due to the social context (i.e., not the strain × context association). Instead, it seems that the attractive nature of the white box, whatever that may be, overpowered any effect of the play partner despite the F344 partners playing less than the LE partner, providing hence a different play experience. Rather, it is the environmental context that facilitated the preferred experiences that is selected and not the partner during the sCPP preference test. In any case, this suggests that rats do prefer contexts in which they had experienced more attractive play experiences. However, this can be driven by the context/enclosure and not necessarily the identity of the partner. Nevertheless, because the rats were isolated for 23.5 h a day for 8 consecutive days, they were highly motivated to play (Panksepp, 1981). After such chronic social isolation, socializing, regardless of the quality or nature of the interaction, may be so rewarding that the possible effect of the identity of the partner was masked. Consequently, the present findings do not preclude the possibility that in a different testing paradigm, rats would show a preference for one strain over another. Even so, as noted above, the focal rats had a higher rate of vocalizing when in both the least preferred black box and in the box, either white or black, that had involved playing with a F344 partner, which suggests that there may be subtle aspects of behavior that reflect preferences, including social ones. Again, this reinforces our earlier point that constructing the appropriate test paradigm and identifying the most appropriate behavioral markers are central to drawing firm conclusions about what is and what is not salient to the animals.

One limitation of this study is that we only used males. Indeed, the appropriate test paradigm and behavioral markers may need to differ for females despite male and female juvenile male rats using the same RTP behavioral patterns (Pellis et al., 2022a). Female rats are more sensitive to the familiarity of their partner when playing in dyads, playing more with familiar animals than strangers (Argue and McCarthy, 2015a), and, similarly, they may be more sensitive to the strain of their play partners. As such, replicating this study with females may further refine the conclusions of this study.

## Conclusion

The use of rat RTP has become increasingly popular as a tool for studying social regulation, communication, and development given that is a naturally occurring behavior in which rats are highly motivated to engage (Pellis et al., 2022b). While it is a powerful tool, our results highlight that the testing paradigm used can greatly affect the behavioral outcomes. For example, when LE rats are given the choice, they prefer to play with same-strain partners. However, this preference is no longer present when the testing paradigm was changed. When tested using the dyadic paradigm (Pellis et al., 2022a), the rats did not play more with same-strain partners. Similarly, focal LE rats did not appear to prefer contexts that were paired with a same-strain partner after 8 days of conditioning using a modified sCPP paradigm (Achterberg et al., 2012, 2019; Trezza et al., 2009, 2011). Instead, the LE rats preferred the context in which they played more, regardless of the identity of the partner. In other words, the environmental or social context in which rats find themselves in may trump partner preferences. In addition to changes in partner preferences, we also found that when playing in groups, the rats played 3–4 times more, suggesting there is a group play contagion effect in rats. Our results underline the need to keep the natural ecology and ethology of the animal in mind when designing studies of play behavior in rats given that the testing paradigm, environment, and social partners used can greatly influence the behavioral outcome.

## Data Availability

The raw data supporting the conclusions of this article will be made available by the authors, without undue reservation.

## References

[B1] Achterberg E. J. M. Vanderschuren L. J. M. J. (2020). Treatment with low doses of nicotine but not alcohol affects social play reward in rats. *Int. J. Play* 9 39–57. 10.1080/21594937.2020.1720121

[B2] Achterberg E. J. M. Vanderschuren L. J. M. J. (2023). The neurobiology of social play behaviour: Past, present and future. *Neurosci. Biobehav. Rev.* 152:105319. 10.1016/j.neubiorev.2023.105319 37454882

[B3] Achterberg E. J. M. Burke C. J. Pellis S. M. (2023). When the individual comes into play: The role of self and the partner in the dyadic play fighting of rats. *Behav. Process.* 12:104933. 10.1016/j.beproc.2023.104933 37643663

[B4] Achterberg E. J. M. Trezza V. Vanderschuren L. J. M. J. (2012). β-Adrenoreceptor stimulation mediates reconsolidation of social reward-related memories. *PLoS One* 7:e39639. 10.1371/journal.pone.0039639 22745800 PMC3379988

[B5] Achterberg E. J. M. Trezza V. Vanderschuren L. J. M. J. (2014). Glucocorticoid receptor antagonism disrupts the reconsolidation of social reward-related memories in rats. *Behav. Pharmacol.* 25 216–225. 10.1097/FBP.0000000000000039 24776489 PMC4006345

[B6] Achterberg E. J. M. van Swieten M. M. H. Driel N. V. Trezza V. Vanderschuren L. J. M. J. (2016). Dissociating the role of endocannabinoids in the pleasurable and motivational properties of social play behaviour in rats. *Pharmacol. Res.* 110 151–158. 10.1016/j.phrs.2016.04.031 27154553 PMC4914428

[B7] Achterberg E. J. M. van Swieten M. M. H. Houwing D. J. Trezza V. Vanderschuren L. J. M. J. (2019). Opioid modulation of social play reward in juvenile rats. *Neuropharmacology* 159:107332. 10.1016/j.neuropharm.2018.09.007 30218673

[B8] Argue K. J. McCarthy M. M. (2015a). Characterization of juvenile play in rats: Importance of sex of self and sex of partner. *Biol. Sex Differ.* 6:16. 10.1186/s13293-015-0034-x 26361539 PMC4564956

[B9] Argue K. J. McCarthy M. M. (2015b). Utilization of same- vs. mixed-sex dyads impacts the observation of sex differences in juvenile social play behavior. *Curr. Neurobiol.* 6 17–23. 10.4172/0975-9042.000106 26924913 PMC4765144

[B10] Barnett S. A. (1958). An analysis of social behaviour in wild rats. *Proc. Zool. Soc. Lond.* 130 107–152. 10.1111/j.1096-3642.1958.tb00565.x

[B11] Bateson M. Martin P. (2021). *Measuring behaviour. An introductory guide*, 4th Edn. Cambridge: Cambridge University Press.

[B12] Bijlsma A. Birza E. E. Pimentel T. C. Maranus J. P. M. Van Gaans M. J. J. M. Lozeman-Van T Klooster J. G. (2024). Opportunities for risk-taking during play alters cognitive performance and prefrontal inhibitory signalling in rats of both sexes. *Eur. J. Neurosci.* 59 2748–2765. 10.1111/ejn.16313 38511534

[B13] Bijlsma A. Omrani A. Spoelder M. Verharen J. P. H. Bauer L. Zwart B. D. (2022). Social play is critical for the development of prefrontal inhibitory synapses and cognitive flexibility in rats. *J. Neurosci.* 42 8716–8728. 10.1523/JNEUROSCI.0524-22.2022 36253083 PMC9671579

[B14] Brudzynski S. (2015). Pharmacology of ultrasonic vocalizations in adult rats: Significance, call classification and neural substrate. *Curr. Neuropharmacol.* 13 180–192. 10.2174/1570159X13999150210141444 26411761 PMC4598430

[B15] Burgdorf J. Kroes R. A. Moskal J. R. Pfaus J. G. Brudzynski S. M. Panksepp J. (2008). Ultrasonic vocalizations of rats (*Rattus norvegicus*) during mating, play, and aggression: Behavioral concomitants, relationship to reward, and self-administration of playback. *J. Comp. Psychol.* 122 357–367. 10.1037/a0012889 19014259

[B16] Burghardt G. M. (2005). *The genesis of animal play: Testing the limits.* Cambridge, MA: MIT Press.

[B17] Burke C. J. Euston D. R. Pellis S. M. (2020). What do you hear, what do you say? Ultrasonic calls as signals during play fighting in rats. *Int. J. Play* 9 92–107. 10.1080/21594937.2020.1720126

[B18] Burke C. J. Kisko T. M. Euston D. R. Pellis S. M. (2018). Do juvenile rats use specific ultrasonic calls to coordinate their social play? *Anim. Behav.* 140 81–92. 10.1016/j.anbehav.2018.03.019

[B19] Burke C. J. Kisko T. M. Swiftwolfe H. Pellis S. M. Euston D. R. (2017). Specific 50-kHz vocalizations are tightly linked to particular types of behavior in juvenile rats anticipating play. *PLoS One* 12:e0175841. 10.1371/journal.pone.0175841 28467436 PMC5414981

[B20] Burke C. J. Modlinska K. Mauro M. H. Aleksandrova L. R. Pellis S. M. Phillips A. G. (2021). A naturalistic method to test depression: Anticipation of play. *Behav. Brain Res.* 398:112975. 10.1016/j.bbr.2020.112975 33141076

[B21] Burke C. J. Pellis S. M. Achterberg E. J. M. (2022). Who’s laughing? Play, tickling and ultrasonic vocalizations in rats. *Philos. Trans. R. Soc. B Biol. Sci.* 377:20210184. 10.1098/rstb.2021.0184 36126668 PMC9489288

[B22] Calhoun J. B. (1963). *The ecology and sociology of the Norway rat.* Bethesda, MD: US Department of Health, Education, and Welfare, Public Health Service, 10.1002/bip

[B23] d’Isa R. Gerlai R. (2023). Designing animal-friendly behavioral tests for neuroscience research: The importance of an ethological approach. *Front. Behav. Neurosci.* 16:1090248. 10.3389/fnbeh.2022.1090248 36703720 PMC9871504

[B24] Dennis E. J. El Hady A. Michaiel A. Clemens A. Tervo D. R. G. Voigts J. (2021). Systems neuroscience of natural nehaviors in rodents. *J. Neurosci.* 41 911–919. 10.1523/JNEUROSCI.1877-20.2020 33443081 PMC7880287

[B25] Fagen R. (1981). *Animal play behavior.* Oxford: Oxford University Press.

[B26] Gaub S. Fisher S. E. Ehret G. (2016). Ultrasonic vocalizations of adult male Foxp2 -mutant mice: Behavioral contexts of arousal and emotion. *Genes Brain Behav.* 15 243–259. 10.1111/gbb.12274 26566793

[B27] Ham J. R. Pellis S. (2023). The Goldilocks principle: Balancing familiarity and novelty in the selection of play partners in groups of juvenile male rats. *Anim. Behav. Cogn.* 10 304–328. 10.26451/abc.10.04.02.2023 39076802

[B28] Ham J. R. Pellis S. M. (2024). Play partner preferences among groups of unfamiliar juvenile male rats. *Sci. Rep.* 14:16056. 10.1038/s41598-024-66988-w 38992171 PMC11239858

[B29] Ham J. R. Pellis S. M. (2025). Groups of familiar male rats form unstable partner preferences when play fighting during the juvenile period. *iScience* 28:112562. 10.1016/j.isci.2025.112562 40487429 PMC12144427

[B30] Ham J. R. Szabo M. Annor-Bediako J. Stark R. A. Iwaniuk A. N. Pellis S. M. (2024). Quality not quantity: Deficient juvenile play experiences lead to altered medial prefrontal cortex neurons and sociocognitive skill deficits. *Dev. Psychobiol.* 66:e22456. 10.1002/dev.22456 38388195

[B31] Himmler B. T. Pellis V. C. Pellis S. M. (2013a). Peering into the dynamics of social interactions: Measuring play fighting in rats. *J. Vis. Exp.* 71 1–8. 10.3791/4288 23353923 PMC3582623

[B32] Himmler B. T. Stryjek R. Modlinska K. Derksen S. M. Pisula W. Pellis S. M. (2013b). How domestication modulates play behavior: A comparative analysis between wild rats and a laboratory strain of *Rattus norvegicus*. *J. Comp. Psychol.* 127 453–464. 10.1037/a0032187 23815592

[B33] Himmler S. M. Himmler B. T. Pellis V. C. Pellis S. M. (2016a). Play, variation in play and the development of socially competent rats. *Behaviour* 153 1103–1137. 10.1163/1568539X-00003307

[B34] Himmler S. M. Himmler B. T. Stryjek R. Modlińska K. Pisula W. Pellis S. M. (2016b). Pinning in the play fighting of rats: A comparative perspective with methodological recommendations. *Int. J. Comp. Psychol.* 29 6. 10.46867/ijcp.2016.29.00.06

[B35] Himmler S. M. Lewis J. M. Pellis S. M. (2014a). The development of strain typical defensive patterns in the play fighting of laboratory rats. *Int. J. Comp. Psychol.* 27 385–396. 10.46867/ijcp.2014.27.03.09

[B36] Himmler S. M. Modlinska K. Stryjek R. Himmler B. T. Pisula W. Pellis S. M. (2014b). Domestication and diversification: A comparative analysis of the play fighting of the Brown Norway, Sprague-Dawley, and Wistar laboratory strains of (*Rattus norvegicus*). *J. Comp. Psychol.* 128 318–327. 10.1037/a0036104 24749500

[B37] Hole G. H. Einon D. F. (1984). “Play in rodents,” in *Play in animals and children*, ed. Smith P. K. (Hoboken, NJ: Blackwell), 95–117.

[B38] Holman P. J. Baglot S. L. Morgan E. Weinberg J. (2019). Effects of prenatal alcohol exposure on social competence: Asymmetry in play partner preference among heterogeneous triads of male and female rats. *Dev. Psychobiol.* 61 513–524. 10.1002/dev.21842 30843198 PMC6531035

[B39] Kisko T. M. Euston D. R. Pellis S. M. (2015). Are 50-khz calls used as play signals in the playful interactions of rats? III. The effects of devocalization on play with unfamiliar partners as juveniles and as adults. *Behav. Process.* 113 113–121. 10.1016/j.beproc.2015.01.016 25643949

[B40] Kiyokawa Y. Kuroda N. Takeuchi Y. (2022). The strain of unfamiliar conspecifics affects stress identification in rats. *Behav. Process.* 201:104714. 10.1016/j.beproc.2022.104714 35901937

[B41] Knutson B. Burgdorf J. Panksepp J. (1998). Anticipation of play elicits high-frequency ultrasonic vocalizations in young rats. *J. Comp. Psychol.* 112 65–73. 10.1037/0735-7036.112.1.65 9528115

[B42] Kogo H. Maeda N. Kiyokawa Y. Takeuchi Y. (2021). Rats do not consider all unfamiliar strains to be equivalent. *Behav. Process.* 190:104457. 10.1016/j.beproc.2021.104457 34216685

[B43] Kondrakiewicz K. Kostecki M. Szadzińska W. Knapska E. (2019). Ecological validity of social interaction tests in rats and mice. *Genes Brain Behav.* 18:e12525. 10.1111/gbb.12525 30311398

[B44] Lampe J. F. Ruchti S. Burman O. Würbel H. Melotti L. (2019). Play like me: Similarity in playfulness promotes social play. *PLoS One* 14:e0224282. 10.1371/journal.pone.0224282 31648250 PMC6812795

[B45] Lesscher H. M. B. Achterberg E. J. M. Siviy S. M. Vanderschuren L. J. M. J. (2021). Individual differences in social play behaviour predict alcohol intake and control over alcohol seeking in rats. *Psychopharmacology* 238 3119–3130. 10.1007/s00213-021-05929-1 34338827 PMC8605978

[B46] Martínez M. Guillén-Salazar F. Salvador A. Simón V. M. (1995). Successful intermale aggression and conditioned place preference in mice. *Physiol. Behav.* 58 323–328. 10.1016/0031-9384(95)00061-M 7568436

[B47] McDonald R. J. Hong N. S. Atwood A. Tyndall A. V. Kolb B. (2021). An assessment of the functional effects of amphetamine-induced dendritic changes in the nucleus accumbens, medial prefrontal cortex, and hippocampus on different types of learning and memory function. *Neurobiol. Learn. Mem.* 180:107408. 10.1016/j.nlm.2021.107408 33609742

[B48] McDonald R. J. Hong N. S. Trow J. S. Kaupp C. Balog R. J. Gokarn L. (2023). Effects of maternal social isolation on adult rodent offspring cognition. *Sci. Rep.* 13:7748. 10.1038/s41598-023-34834-0 37173349 PMC10177704

[B49] Meaney M. J. (1988). The sexual differentiation of social play. *Trends Neurosci.* 11 54–58. 10.1016/0166-2236(88)90164-6 2465599

[B50] Meaney M. J. Stewart J. (1981). A descriptive study of social development in the rat (*Rattus norvegicus*). *Anim. Behav.* 29 34–45. 10.1016/S0003-3472(81)80149-2

[B51] Northcutt K. V. Nwankwo V. C. (2018). Sex differences in juvenile play behavior differ among rat strains. *Dev. Psychobiol.* 60 903–912. 10.1002/DEV.21760 29969514

[B52] Orsucci I. C. Becker K. D. Ham J. R. Lee J. D. A. Bowden S. M. Veenema A. H. (2024). To play or not to play? Effects of playmate familiarity and social isolation on social play engagement in three laboratory rat strains. *bioRxiv* [Preprint]. 2024:623692. 10.1101/2024.11.14.623692 39605718 PMC11601367

[B53] Panksepp J. (1981). The ontogeny of play in rats. *Dev. Psychobiol.* 14 327–332. 10.1002/dev.420140405 7250521

[B54] Pellis S. M. (2002). Sex differences in play fighting revisited: Traditional and nontraditional mechanisms of sexual differentiation in rats. *Arch. Sex. Behav.* 31 17–26. 10.1023/A:1014070916047 11910788

[B55] Pellis S. M. Pellis V. C. (1987). Play-fighting differs from serious fighting in both target of attack and tactics of fighting in the laboratory rat *Rattus norvegicus*. *Aggress. Behav.* 13 227–242. 10.1002/1098-2337198713:4<227::AID-AB2480130406<3.0.CO;2-C

[B56] Pellis S. M. Pellis V. C. (1990). Differential rates of attack, defense, and counterattack during the developmental decrease in play fighting by male and female rats. *Dev. Psychobiol.* 23 215–231. 10.1002/dev.420230303 2379760

[B57] Pellis S. M. Pellis V. C. (1997). The prejuvenile onset of play fighting in laboratory rats (*Rattus norvegicus*). *Dev. Psychobiol.: J. Int. Soc. Dev. Psychobiol.* 31, 193–205. 10.1002/(SICI)1098-2302(199711)31:3<193::AID-DEV4>3.0.CO;2-N 9386921

[B58] Pellis S. M. Pellis V. C. (1998). “Structure–function interface in the analysis of play,” in *Animal play: Evolutionary, comparative, and ecological perspectives*, eds Bekoff M. Byers J. A. (Cambridge: Cambridge University Press), 115–140.

[B59] Pellis S. M. Pellis V. C. (2009). *The playful brain: Venturing to the limits of neuroscience.* London: Oneworld Publications.

[B60] Pellis S. M. Pellis V. C. (2021). *Understanding animal behaviour: What to measure and why.* Cambridge: Cambridge University Press.

[B61] Pellis S. M. Pellis V. C. Ham J. R. (2024). Play fighting revisited: Its design features and how they shape our understanding of its mechanisms and functions. *Front. Ethol.* 3:1362052. 10.3389/fetho.2024.1362052

[B62] Pellis S. M. Pellis V. C. Burke C. J. Stark R. A. Ham J. R. Euston D. R. (2022a). Measuring play fighting in rats: A multilayered approach. *Curr. Protoc.* 2:e337. 10.1002/cpz1.337 35030300

[B63] Pellis S. M. Pellis V. C. Ham J. R. Achterberg E. J. M. (2022b). The rough-and-tumble play of rats as a natural behavior suitable for studying the social brain. *Front. Behav. Neurosci.* 16:1033999. 10.3389/fnbeh.2022.1033999 36330048 PMC9623181

[B64] Pellis S. M. Pellis V. C. Ham J. R. Stark R. A. (2023). Play fighting and the development of the social brain: The rat’s tale. *Neurosci. Biobehav. Rev.* 145:105037. 10.1016/j.neubiorev.2023.105037 36621585

[B65] Raza S. Himmler B. T. Himmler S. M. Harker A. Kolb B. Pellis S. M. (2015). Effects of prenatal exposure to valproic acid on the development of juvenile-typical social play in rats. *Behav. Pharmacol.* 26 707–719. 10.1097/FBP.0000000000000169 26230723

[B66] Reinhart C. J. McIntyre D. C. Metz G. A. Pellis S. M. (2006). Play fighting between kindling-prone (FAST) and kindling-resistant (SLOW) rats. *J. Comp. Psychol.* 120 19–30. 10.1037/0735-7036.120.1.19 16551161

[B67] Reinhold A. S. Sanguinetti-Scheck J. I. Hartmann K. Brecht M. (2019). Behavioral and neural correlates of hide-and-seek in rats. *Science* 365 1180–1183. 10.1126/science.aax4705 31515395

[B68] Roux S. Froger C. Porsolt R. D. Valverde O. Maldonado R. (2002). Place Preference Test in Rodents. *Curr. Protoc. Pharmacol.* 19:19. 10.1002/0471141755.ph1004s19 22294083

[B69] Schneider P. Bindila L. Schmahl C. Bohus M. Meyer-Lindenberg A. Lutz B. (2016). Adverse social experiences in adolescent rats result in enduring effects on social competence, pain sensitivity and endocannabinoid signaling. *Front. Behav. Neurosci.* 10:203. 10.3389/fnbeh.2016.00203 27812328 PMC5071316

[B70] Schweinfurth M. K. (2020). The social life of Norway rats (*Rattus norvegicus*). *Elife* 9:e54020. 10.7554/eLife.54020 32271713 PMC7145424

[B71] Siviy S. M. (2016). A brain motivated to play: Insights into the neurobiology of playfulness. *Behaviour* 153 819–844. 10.1163/1568539X-00003349 29056751 PMC5646690

[B72] Siviy S. M. Crawford C. A. Akopian G. Walsh J. P. (2011). Dysfunctional play and dopamine physiology in the Fischer 344 rat. *Behav. Brain Res.* 220, 294–304. 10.1016/j.bbr.2011.02.009 21335036 PMC3081852

[B73] Siviy S. M. Panksepp J. (1987). Sensory modulation of juvenile play in rats. *Dev. Psychobiol.* 20 39–55. 10.1002/dev.420200108 3556783

[B74] Siviy S. M. Baliko C. N. Bowers K. S. (1997). Rough-and-tumble play behavior in Fischer-344 and Buffalo rats: Effects of social isolation. *Physiol. Behav.* 61 597–602. 10.1016/S0031-9384(96)00509-4 9108580

[B75] Siviy S. M. Eck S. R. McDowell L. S. Soroka J. (2017). Effects of cross-fostering on play and anxiety in juvenile Fischer 344 and Lewis rats. *Physiol. Behav.* 169 147–154. 10.1016/j.physbeh.2016.11.035 27923716 PMC5183544

[B76] Siviy S. M. Love N. J. DeCicco B. M. Giordano S. B. Seifert T. L. (2003). The relative playfulness of juvenile Lewis and Fischer-344 rats. *Physiol. Behav.* 80 385–394. 10.1016/j.physbeh.2003.09.002 14637239

[B77] Siviy S. M. Martin M. A. Campbell C. M. (2023). Noradrenergic modulation of play in Sprague-Dawley and F344 rats. *Psychopharmacology* 242 955–964. 10.1007/s00213-023-06419-2 37428218

[B78] Stark R. A. Pellis S. M. (2020). Male long Evans rats reared with a Fischer-344 peer during the juvenile period show deficits in social competency: A role for play. *Int. J. Play* 9 76–91. 10.1080/21594937.2020.1720142

[B79] Stark R. A. Pellis S. M. (2021). Using the ‘stranger test’ to assess social competency in adult female Long Evans rats reared with a Fischer 344 partner. *Behav. Process.* 192:104492. 10.1016/j.beproc.2021.104492 34478804

[B80] Stark R. A. Ramkumar R. Pellis S. M. (2021). Deficient play-derived experiences in juvenile Long Evans rats reared with a Fischer 344 partner: A deficiency shared by both sexes. *Int. J. Comp. Psychol.* 34 1–19. 10.46867/ijcp.2021.34.5592

[B81] Thor D. H. Holloway W. R. (1983). Play-solicitation behavior in juvenile male and female rats. *Anim. Learn. Behav.* 11 173–178. 10.3758/BF03199645

[B82] Thor D. H. Holloway W. R. (1984a). Developmental analyses of social play behavior in juvenile rats. *Bull. Psychon. Soc.* 22 587–590. 10.3758/BF03333916

[B83] Thor D. H. Holloway W. R. (1984b). Social play in juvenile rats: A decade of methodological and experimental research. *Neurosci. Biobehav. Rev.* 8 455–464. 10.1016/0149-7634(84)90004-6 6514252

[B84] Trezza V. Vanderschuren L. J. M. J. (2008). Cannabinoid and opioid modulation of social play behavior in adolescent rats: Differential behavioral mechanisms. *Eur. Neuropsychopharmacol.* 18 519–530. 10.1016/j.euroneuro.2008.03.001 18434104 PMC2490798

[B85] Trezza V. Damsteegt R. Vanderschuren L. J. M. J. (2009). Conditioned place preference induced by social play behavior: Parametrics, extinction, reinstatement and disruption by methylphenidate. *Eur. Neuropsychopharmacol.* 19 659–669. 10.1016/j.euroneuro.2009.03.006 19427175 PMC2716414

[B86] Trezza V. Damsteegt R. Marijke Achterberg E. J. Vanderschuren L. J. M. J. (2011). Nucleus accumbens μ-opioid receptors mediate social reward. *J. Neurosci.* 31 6362–6370. 10.1523/JNEUROSCI.5492-10.2011 21525276 PMC3098965

[B87] Vanderschuren L. J. M. J. (2010). How the brain makes play fun. *Am. J. Play* 2 315–337.

[B88] VanRyzin J. W. Marquardt A. E. Argue K. J. Vecchiarelli H. A. Ashton S. E. Arambula S. E. (2019). Microglial phagocytosis of newborn cells is induced by endocannabinoids and sculpts sex differences in juvenile rat social play. *Neuron* 102:435–449.e6. 10.1016/j.neuron.2019.02.006 30827729 PMC8046232

[B89] Veenema A. H. Bredewold R. De Vries G. J. (2013). Sex-specific modulation of juvenile social play by vasopressin. *Psychoneuroendocrinology* 38 2554–2561. 10.1016/j.psyneuen.2013.06.002 23838102 PMC3812261

[B90] Wöhr M. Houx B. Schwarting R. K. W. Spruijt B. (2008). Effects of experience and context on 50-kHz vocalizations in rats. *Physiol. Behav.* 93 766–776. 10.1016/j.physbeh.2007.11.031 18191963

[B91] Wright J. M. Gourdon J. C. Clarke P. B. S. (2010). Identification of multiple call categories within the rich repertoire of adult rat 50-kHz ultrasonic vocalizations: Effects of amphetamine and social context. *Psychopharmacology* 211 1–13. 10.1007/s00213-010-1859-y 20443111

